# ﻿Two new cave *Hyleoglomeris* species (Glomerida, Glomeridae) from northern Vietnam

**DOI:** 10.3897/zookeys.1108.85423

**Published:** 2022-06-24

**Authors:** Mai Kuroda, Katsuyuki Eguchi, Emiko Oguri, Anh D. Nguyen

**Affiliations:** 1 Faculty of Education, Tokyo Gakugei University, 4-chome-1-1 Nukuikitamachi, Koganei Tokyo, 184-8501, Japan Tokyo Gakugei University Tokyo Japan; 2 Graduate School of Science, Tokyo Metropolitan University, Minami-osawa 1-1, Hachioji, Tokyo 192-0397, Japan Tokyo Metropolitan University Tokyo Japan; 3 Department of International Health and Medical Anthropology, Institute of Tropical Medicine, 1-12-4 Sakamoto, Nagasaki University, Nagasaki, 852-8523, Japan Nagasaki University Nagasaki Japan; 4 Department of Soil Ecology, Institute of Ecology and Biological Resources, Vietnam Academy of Science and Technology, 18, Hoangquocviet Rd., Caugiay District, Hanoi, Vietnam Institute of Ecology and Biological Resources, Vietnam Academy of Science and Technology Hanoi Vietnam; 5 Graduate University of Science and Technology, Vietnam Academy of Science and Technology, 18, Hoangquocviet Rd., Caugiay District, Hanoi, Vietnam Graduate University of Science and Technology Hanoi Vietnam

**Keywords:** Biodiversity, millipede, new species, taxonomy, troglobiont

## Abstract

Two new glomerid species from caves in Cao Bang Province, Northern Vietnam, namely, *Hyleoglomerishalang* Kuroda, Nguyen & Eguchi, **sp. nov.** and *Hyleoglomerisalba* Nguyen, Kuroda & Eguchi, **sp. nov.**, are described. The former is characterized by a distinct body color pattern; telopods with a large, quadrate, medially concave, sparsely setose, central syncoxital lobe; and syncoxital horns approximately 1.5–2.0 times as long as the lobe. The latter is distinguished by its completely troglobiotic form without eyes, an unpigmented body, and a roundly triangular syncoxital lobe of telopods. An identification key is also provided for the cave glomerids of Vietnam.

## ﻿Introduction

Currently, 23 glomerid species in six genera (*Annameris* Verhoeff, 1915, *Hyleoglomeris* Verhoeff, 1910, *Hyperglomeris* Silvestri, 1917, *Peplomeris* Silvestri, 1917, *Rhopalomeris* Verhoeff, 1906, and *Tonkinomeris* Nguyen, Sierwald & Marek, 2019) have been recorded and described from Vietnam (Nguyen et al. 2019a, b, 2021). Of these species, five were described from caves, *Hyleoglomeriscavernicola* Golovatch, Geoffroy & VandenSpiegel, 2013 and *Hyleoglomerisspeophila* Golovatch, Geoffroy & Mauriès, 2006, both from Cat Ba National Park; *Hyleoglomeriscolorata* Golovatch, Geoffroy & VandenSpiegel, 2013 and *Hyleoglomerisspelaea* Golovatch, Geoffroy & VandenSpiegel, 2013 both from Phong Nha - Ke Bang National Park; and *Hyperglomerisdepigmentata* Golovatch, Geoffroy & VandenSpiegel, 2013 from Thanh Hoa Province (Golovatch et al. 2006, 2013).

Of the six genera, *Hyleoglomeris* is a rich-species genus, not only in Vietnam (12 species; Nguyen et al. 2019a, 2019b) but also worldwide (~ 100 species) (Golovatch et al. 2006; Wesener 2015; Sierwald and Spelda 2021). The genus is widely distributed from Greece in the west, Japan in the east, and Sulawesi (Indonesia) in the southeast (Golovatch et al. 2006). Therefore, it is not surprising that most Vietnamese cave glomerids belong to this genus.

During our field expeditions in northern Vietnam, glomerid specimens that could not be assigned to the named species were discovered and collected. They were both collected from caves and described in this paper.

## ﻿Materials and methods

Specimens were collected manually and directly preserved in 85%–90% ethanol and examined under an Olympus SZX16 microscope. Telopods were dissected for morphological examination and photographed. Colored images were taken using a Nikon SMZ800N microscope and NIS-Element BR v. 5.20.00 and stacked using Helicon Focus v. 7.0. Images were assembled into plates using Photoshop CS6. Terminology follows Golovatch et al. (2013) and Nguyen et al. (2019a).

Total DNA was extracted using Qiagen DNeasy Blood and Tissue Kits. A 680-bp fragment of the mitochondrial gene, cytochrome C oxidase subunit I (COI), was amplified and sequenced using a pair of universal primers, LCO1490 and HCO2198 (Folmer et al. 1994). Polymerase chain reaction (PCR) conditions for amplification of the COI gene follow those of Nguyen et al. (2019b). ExoSap IT was used to successfully purify amplified PCR products, which were then sent for sequencing to the GenLab Company (Hanoi, Vietnam) and the Systematic Zoology Laboratory of Tokyo Metropolitan University (Tokyo, Japan). COI sequences were checked and confirmed using BLASTN 2.6.0+ search (Zhang et al. 2000) and deposited in GenBank.

The holotype, paratypes, and DNA vouchers were preserved in 90% ethanol and deposited at the Institute of Ecology and Biological Resources (**IEBR**), Hanoi, Vietnam.

## ﻿Results

### ﻿Taxonomy

**Order GLOMERIDA**

**Family GLOMERIDAE Leach, 1815**

**Subfamily Doderiinae Slivestri, 1904**

**Genus *Hyleoglomeris* Verhoeff, 1910**

#### ﻿ Hyleoglomeris halang

Taxon classificationAnimaliaGlomeridaGlomeridae

Kuroda, Nguyen & Eguchi sp. nov.

2B6FD3D5-2291-59F0-A245-2FD410E545D2

https://zoobank.org/91A73C3E-10AC-4273-9757-1996DCFBC138

**Figure 1.**
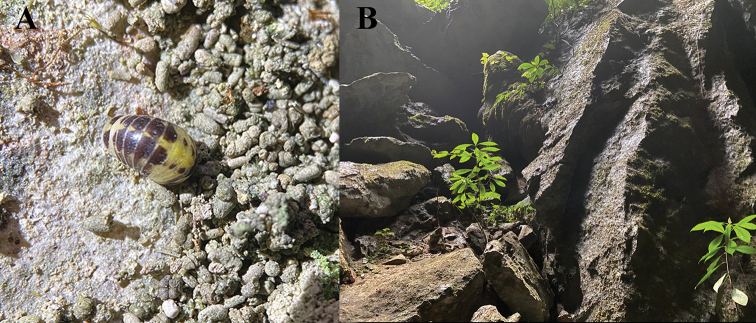
**A***Hyleoglomerishalang* Kuroda, Nguyen & Eguchi, sp. nov. **B** Nguom Hang cave, entrance zone. Images not to scale.

**Figure 2.**
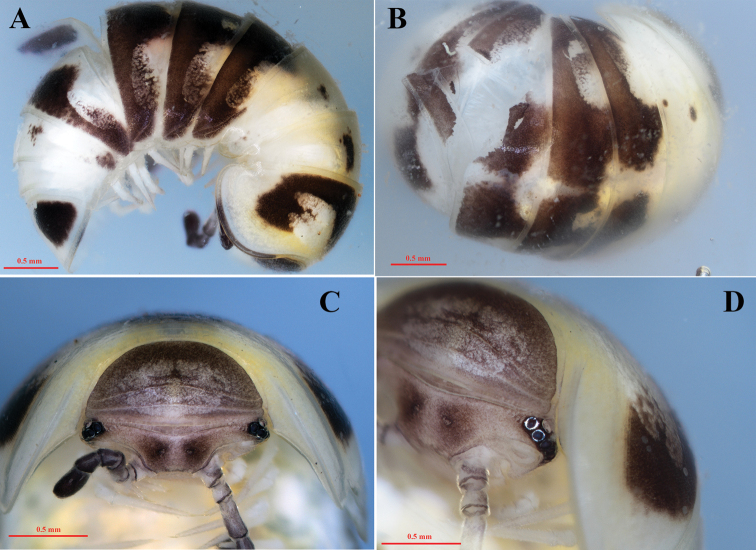
*Hyleoglomerishalang* Kuroda, Nguyen & Eguchi, sp. nov., holotype **A** whole body, lateral view, **B** dorsal view **C** collum, anterior view **D** ocelli, sub-anterior view.

**Figure 3.**
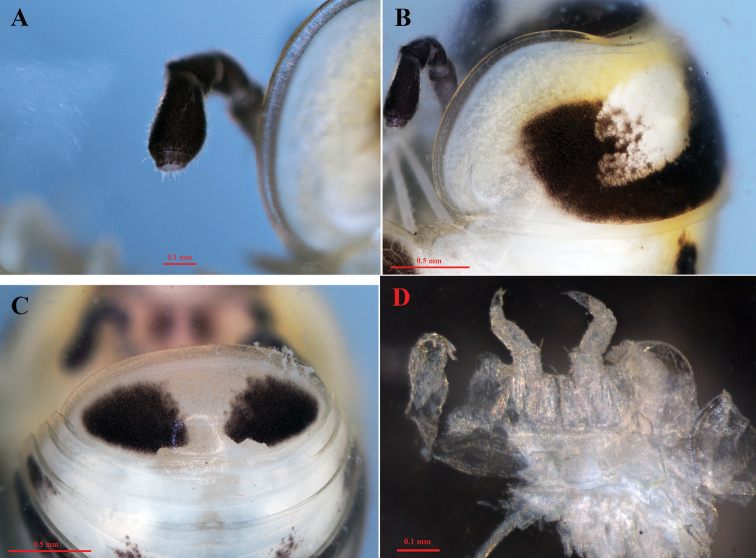
*Hyleoglomerishalang* Kuroda, Nguyen & Eguchi, sp. nov., holotype **A** right antenna **B** hyposchism **C** pygidium, posterior view **D** leg-pair 17.

**Figure 4.**
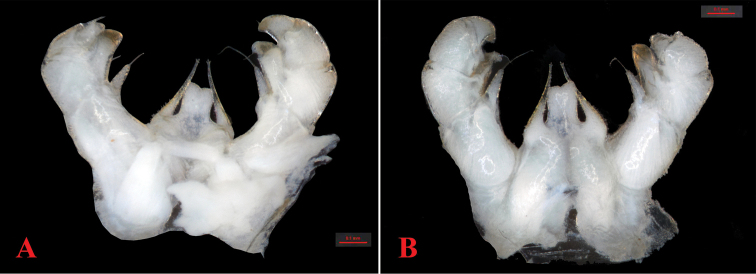
*Hyleoglomerishalang* Kuroda, Nguyen & Eguchi, sp. nov., holotype **A** telopods, anterior view **B** telopods, posterior view.

**Figure 5.**
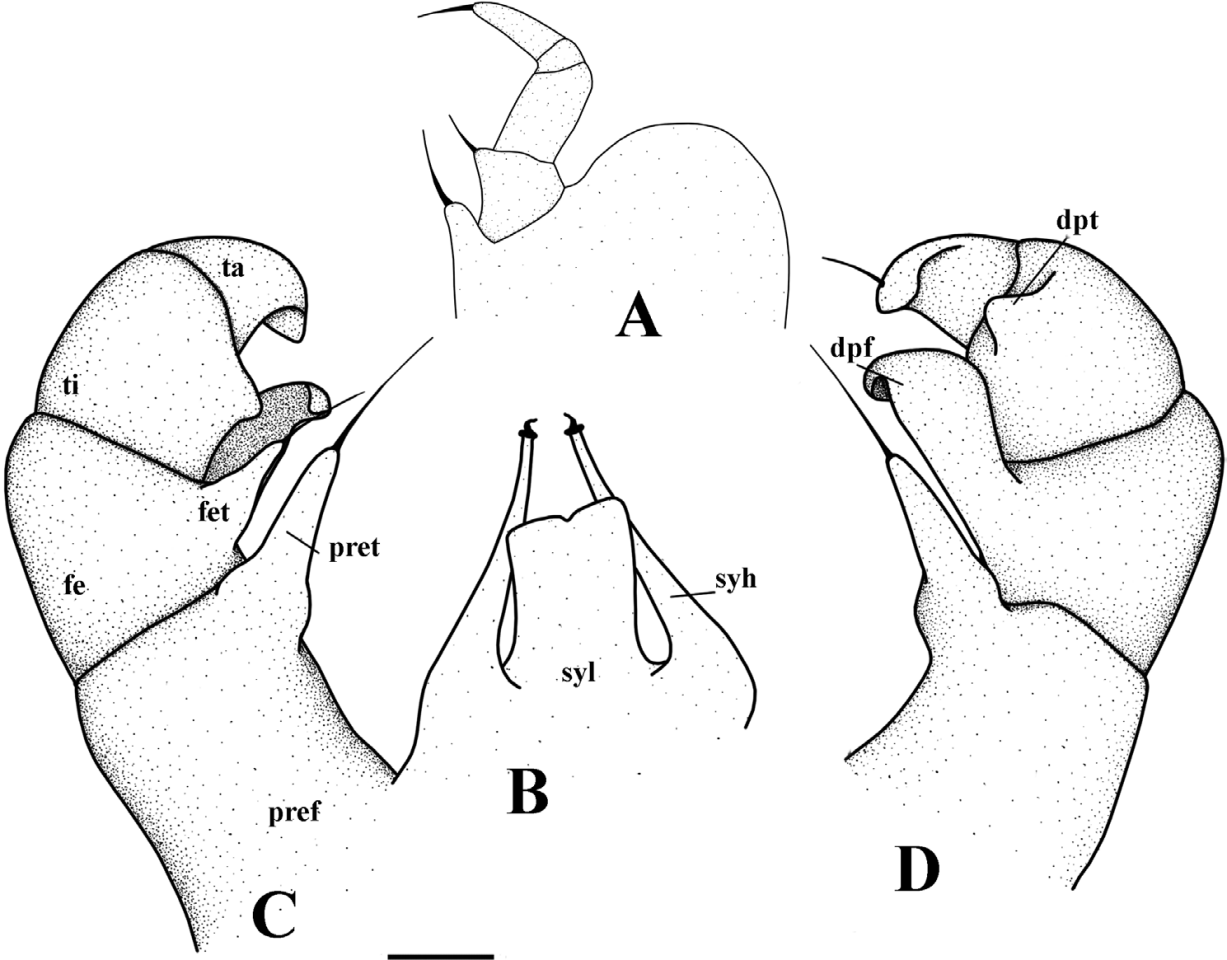
*Hyleoglomerishalang* Kuroda, Nguyen & Eguchi, sp. nov., holotype **A** leg-pair 17 **B** syncoxital lobe and syncoxital horns, posterior view **C** left telopod, posterior view **D** left telopod, anterior view. Scale bar: 0.1 mm. Abbreviations: syl = syncoxital lobe, syh = syncoxital horn, pref = prefemur, fe = femur, pret = prefemoral trichostele, fet = femoral trichostele, dpf = distomesal process of femur, ti = tibia, dpt = distolateral process of tibia, ta = tarsus.

Figs 1 , 2 , 3 , 4 , 5

##### Material examined.

***Holotype*.** Vietnam: male, Cao Bang Province, Ha Lang District, Duc Quang commune, Quang Hoai village, Nguom Hang cave, 22.7208N, 106.6692E, 10 Oct 2020, coll. AD Nguyen, VD Dang & VT Mai (IEBR-Myr 898H). ***Paratypes*.** Vietnam: 1 male, 1 female; Cao Bang Province, Ha Lang District, Duc Quang commune, Quang Hoai village, Nguom Hang cave, 22.7208N, 106.6692E, 10 Oct 2020, coll. AD Nguyen, VD Dang & VT Mai (IEBR-Myr 898P); 1 male, 1 female; Cao Bang Province, Ha Lang District, Duc Quang commune, Quang Hoai village, Nguom Hang cave, 22.7208N, 106.6692E, 15 March 2022, coll. AD Nguyen & DD Nguyen (IEBR-Myr 926).

##### Diagnosis.

The species differs from its congeners in having a distinct body color pattern of white with oval-black spots on terga 4 and 5 and tadpole-shaped black bands on the thoracic shield and terga 6–9; telopods with a large, square/rectangular, slightly concave medially, sparsely setose, central syncoxital lobe; and syncoxital horns ~ 1.5 × as long as the lobe.

The new species can be keyed out at the 26^th^ node in Golovatch et al. (2006), characterized by a thoracic shield and pygidium that are entirely or mainly light and whitish to brown, as well as spots or markings, when present, that is darker than the background. Thus, this species can be grouped with *Hyleoglomeristriangulifera* Attems, 1938 and *Hyleoglomerissiamensis* (Silvestri, 1917). However, its smaller size distinguishes it from both species (3.5 mm wide vs. 6.5 mm and 5.0 mm wide).

##### Etymology.

The new species is named after the Ha Lang District where the types were found. Noun in apposition.

##### Description.

Body length 6 mm, width of the second segment ca. 3.5 mm. Coloration shown as in Figs 2 and 3. Generally white with a line of symmetrical, marbled, black, oval spots at terga 4 and 5, tadpole-shaped black bands on thoracic shield and terga 6–9. Anal shield white, with two laterally symmetrical triangular black spots.

***Head*.** Ocelli 6+1; lenses convex, black contrasting against to a pale blackish background of the head. Tömösváry’s organs transverse and strongly horseshoe-shaped, ~ 2 × as wide and long. Antennae clavate apically; antennomere 6 large, ~ 2.5 × longer than wide; antennal tip with four large, apical sensory cones.

Collum semi-circular, with a very large marbled white spot in the center and two transverse striae; the other parts of the collum pale black. The thoracic shield has a narrow hyposchism, not reaching the caudal margin, with 12 superficial transverse striae, eight of which cross the dorsum.

Leg-pair 17 strongly reduced, 4-segmented with a high, regularly rounded, outer coxal lobe (Figs 3D, 5A). Leg-pair 18 also reduced, but more developed in comparison with leg-pair 17, 4-segmented with a simple V-shaped syncoxital notch.

Telopods (Figs 4, 5B–D) with a large, quadrate, slightly concave medially, sparsely setose, central syncoxital lobe (syl) accompanying two setiferous horns (syh), each directed subventrad, 1.5 × longer than the syncoxital lobe, tip crowned with an apical setoid. Prefemur (pre) and femur (fe) with long trichosteles (pret and fet), prefemoral one (pret) longer than femoral one (fet). Prefemur without additional processes. Distomesal process of femur (dpf) large, long, straight, lamelliform; distal part tuberculiform and strongly curved down, directed laterodorsad. Tibia (ti) with a shorter triangular distolateral process (dpt). Tarsus (ta) slightly sigmoid anteriomesad, subacuminate apically with a seta distoventrally.

##### Variability.

Syncoxital horns can be 2.0 × longer than the syncoxital lobe, each horn directed ventromesad. Two trichosteles are almost of the same in length.

##### DNA barcode.

The COI barcode data (679 bp fragment of the COI) for the paratype was uploaded to GenBank under the accession numbers ON704753 and ON704754. The new species shares 86.98% and 85.89% of its identity with *Hyleoglomerislobus* Nguyen, Sierwald & Marek, 2019 (MT749402) and *Hyleoglomerishoanglien* Nguyen, Eguchi & Hwang, 2019 (MH248038), respectively.

##### Remarks.

This species is not a true cave inhabitant. However, it was discovered in the cave entrance (Fig. 1), and its body is less pigmented with large white areas, suggesting that this species is adapting to a cave-dwelling life.

#### ﻿ Hyleoglomeris alba

Taxon classificationAnimaliaGlomeridaGlomeridae

Nguyen, Kuroda & Eguchi sp. nov.

ED14ECDE-65F8-52FB-ABC1-2D9997C01BFF

https://zoobank.org/C27C76BA-7AD6-4B59-AFE0-6BD5AC9F831F

**Figure 6.**
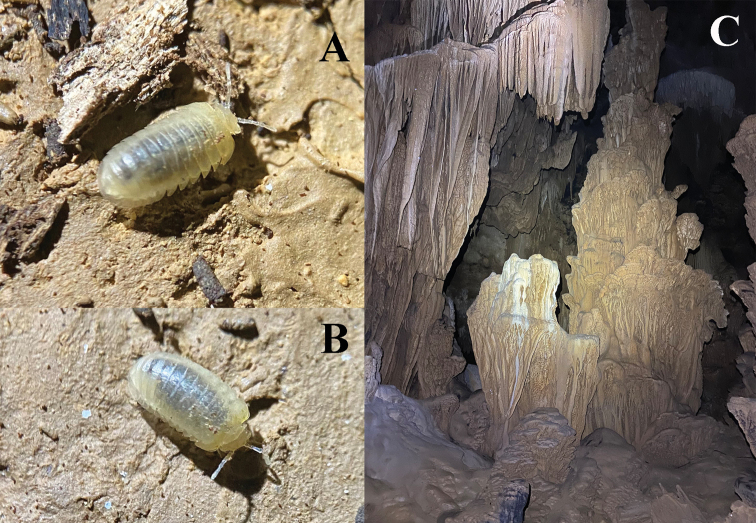
**A, B***Hyleoglomerisalba* Nguyen, Kuroda & Eguchi, sp. nov., habitus **C** Ky Rang cave, dark zone. Images not to scale.

**Figure 7.**
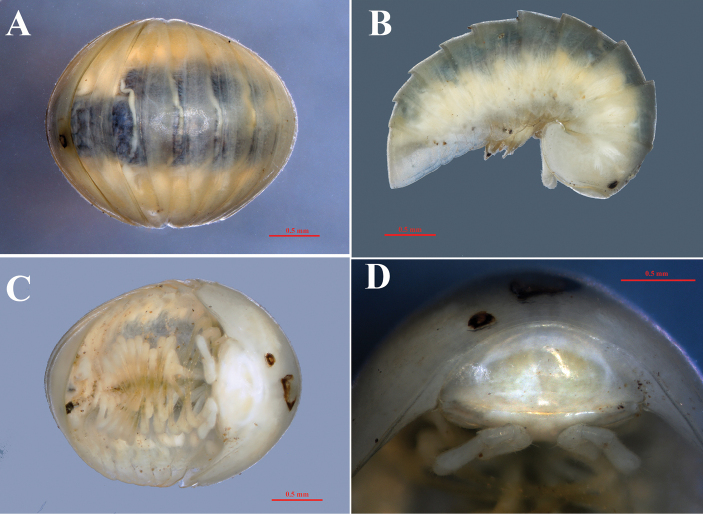
*Hyleoglomerisalba* Nguyen, Kuroda & Eguchi, sp. nov., holotype **A** whole body, dorsal view **B** lateral view **C** ventral view **D** collum, anterior view.

**Figure 8.**
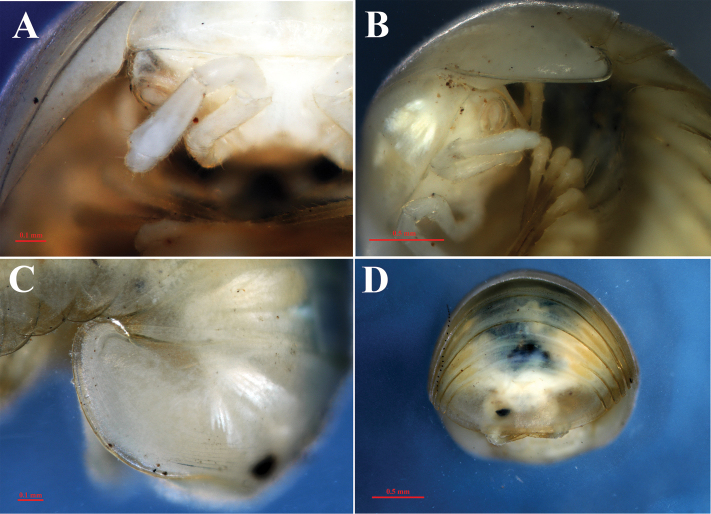
*Hyleoglomerisalba* Nguyen, Kuroda & Eguchi, sp. nov., holotype **A** left antenna **B** right antenna and Tömösváry’s organ **C** left hyposchism, dorsal view **D** pygidium, posterior view.

**Figure 9.**
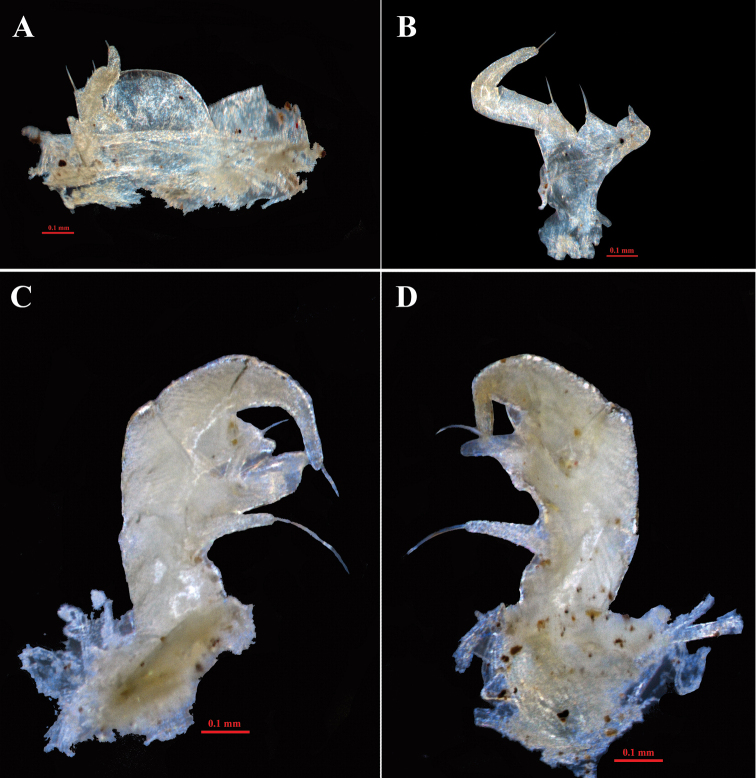
*Hyleoglomerisalba* Nguyen, Kuroda & Eguchi, sp. nov., holotype **A** leg-pair 17 **B** leg 18 **C** left telopod, anterior view **D** subposterior view.

**Figure 10.**
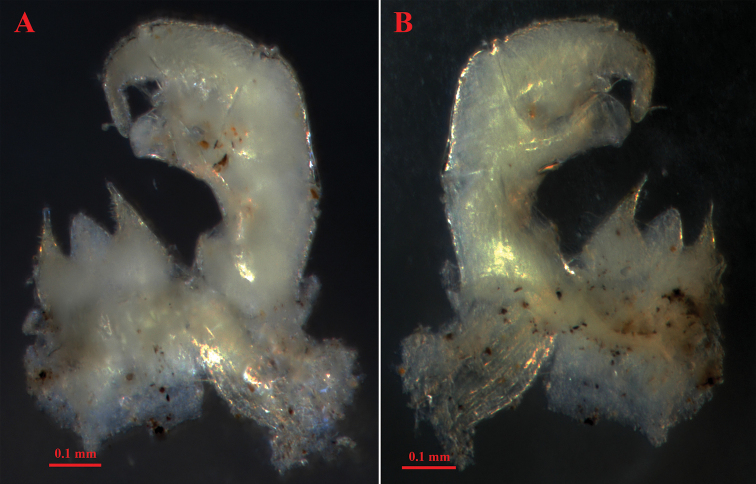
*Hyleoglomerisalba* Nguyen, Kuroda & Eguchi, sp. nov., holotype **A** right telopod and syncoxital lobe, posterior view **B** anterior view.

**Figure 11.**
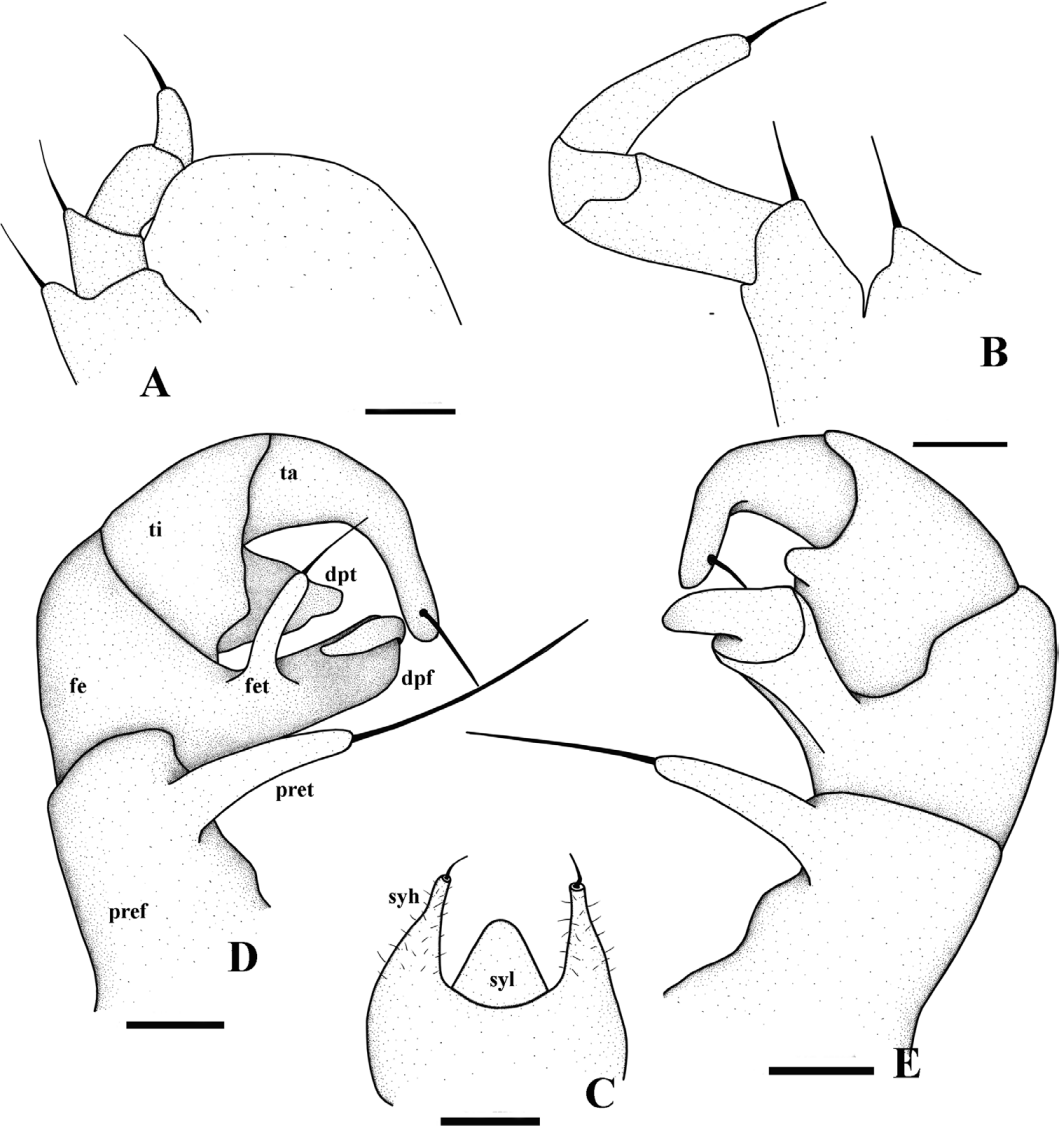
*Hyleoglomerisalba* Nguyen, Kuroda & Eguchi, sp. nov., holotype **A** leg-pair 17 **B** leg-pair 18 **C** syncoxital lobe and syncoxital horns **D** left telopod, anterior view **E** subposterior view. Scale bar: 0.1 mm. Abbreviations: syl = syncoxital lobe, syh = syncoxital horn, pref = prefemur, fe = femur, pret = prefemoral trichostele, fet = femoral trichostele, dpf = distomesal process of femur, ti = tibia, dpt = distolateral process of tibia, ta = tarsus.

Figs 6 , 7 , 8 , 9 , 10 , 11

##### Material examined.

***Holotype*.** Vietnam: male; Cao Bang Province, Tra Linh District, Quoc Toan commune, Thang Hen lake, Ky Rang cave, 22.7650N, 106.2911E, 2 Nov. 2021, leg. AD Nguyen (IEBR-Myr 919). ***Paratypes*.** Vietnam: 2 females, Cao Bang Province, Tra Linh District, Quoc Toan commune, Thang Hen lake, Ky Rang cave, 22.7650N, 106.2911E, 12 Oct. 2020, leg. AD Nguyen, VT Mai & VD Dang (IEBR-Myr 917); 1 male, Cao Bang Province, Tra Linh District, Quoc Toan commune, Thang Hen lake, Ky Rang cave, 22.7650N, 106.2911E, 17 March 2022, leg. AD Nguyen & DD Nguyen (IEBR-Myr 928).

##### Diagnosis.

The species can be recognized by a completely troglobiotic form with no eyes, an unpigmented body, and a roundly triangular syncoxital lobe.

According to Golovatch et al. (2006, 2013), the new species seems to belong to the troglobiont species group containing *H.speophila*, *H.spelaea*, *H.cavernicola*, *H.differens* Golovatch, Geoffroy & Mauriès, 2006, *H.reducta* Golovatch, Geoffroy & Mauriès, 2006, and *H.albicorporis* Zhang & Zhang, 1995. These species are characterized by a totally unpigmented body. However, the new species differs from these species in lacking ocelli and the telopods bearing a roundly triangular syncoxital lobe. In contrast, the other species have convex ocelli, telopods with a roundly subtraperziform syncoxital lobe (*H.speophila*, *H.cavernicola*, *H.reducta*, *H.albicorporis*), or a subquadrate syncoxital lobe (*H.spelaea*), or a roundly triangular syncoxital lobe (*H.differens*).

##### Etymology.

From the Latin *alba*, meaning white. It was used to emphasize the unpigmented body of the new species.

##### Description.

Body length 4.38 mm, width of the second segment ~ 2.02 mm. Color entirely white, unpigmented (Figs 6–8). Ocelli totally absent. TÖmÖsvary’s organ transverse, strongly horseshoe-shaped, ~ 2 × as wide as long (Fig. 8B). Antennae long and slender, antennomere, ~ 3 × longer than wide, antennal tip with four apical sensory cones (Fig. 8A).

Collum semicircular, with a trace of a transverse oval spot in the center and two distinctly transverse striae (Fig. 7D). Second tergum with a narrow hyposchism, not reaching the caudal margin, with seven or eight striae, five or six of which cross the dorsum. Anal shield rounded, very slightly concave medio-caudally (Fig. 8D).

Leg-pair 17 (Figs 9A, 11A) strongly reduced, with four podomeres, with a high, regularly rounded, outer coxal lobe; coxa with an apical setiferous spine; leg-pair 18 (Figs 9B, 11B) also strongly reduced, but more developed in comparison with leg-pair 17, with four podomeres, and a simple V-shaped syncoxital notch.

Telopods (Figs 9C, D, 10, 11C–E) with a roundly triangular, sparsely setose, central syncoxital lobe (syl) accompanying two setiferous syncoxital horns (syh), each directed ventrad, slightly longer than the syncoxital lobe, tip crowned with an apical setoid. Prefemur (pre) and femur (fe) with long trichosteles (pret and fet), prefemoral one (pret) longer than femoral one (fet). Prefemur without additional processes. Distomesal process of femur (dpf) large, long, straight, rectangular; distal part tuberculiform and strongly curved downwards, directed laterodorsad. Tibia (ti) with a shorter triangular distolateral process (dpt). Tarsus (ta) slightly sigmoid anteriomesad, subacuminate apically, with a seta distoventrally.

##### DNA barcode.

We failed to amplify the COI fragments of this species.

##### Remarks.

The species was collected from the totally dark region in the cave. The completely unpigmented body without ocelli, and with long, slender antennae suggest that this species is a true troglobiont. These characters were also mentioned in Liu et al. (2017) who reviewed the morphological adaptations seen in troglobitic glomerids and other millipedes.

### ﻿An identification key to cave glomerids in Vietnam

**Table:** 

1	Prefemoral and femoral trichosteles of telopods absent or rudimentary. Leg-pair 18 3-segmented	***Hyperglomerisdepigmentata***
–	Prefemoral and femoral trichosteles of telopods present, well-developed. Leg-pair 18 4-segmented	**2**
2	Leg-pair 17 3-segmented. Body pattern peculiar, annulated	***Hyleoglomeriscolorata***
–	Leg-pair 17 4-segmented. Body pattern with some dark spots or entirely unpigmented	**3**
3	Body with dark spots	***Hyleoglomerishalang* sp. nov.**
–	Body coloration completely white or unpigmented	**4**
4	Ocelli completely absent. Syncoxital lobe of telopods roundly triangular	***Hyleoglomerisalba* sp. nov.**
–	Ocelli present, sometime poorly visible. Syncoxital lobe of telopods differently shaped but not triangular	**5**
5	Body size small, up to 6 mm long. Syncoxital lobe subquadrate; horn tip with a minute, elongate lobule with a flagelloid filament	***Hyleoglomerisspelaea***
–	Body size larger, 8.5–10 mm. Syncoxital lobe slightly concave or roundly subtrapeziform	**6**
6	Body length 9–10 mm. Syncoxital lobe slightly concave, broadly subtrapeziform; lateral horns simple, unarmed	***Hyleoglomeriscavernicola***
–	Body length 8.5 mm. Syncoxital lobe rounded subtrapeziform; lateral horns each crowned with an apical setoid	***Hyleoglomerisspeophila***

## ﻿General discussion

The northeastern part of the present Indochinese peninsula was covered with a shallow sea from the Late Devonian to the Early Triassic periods (370–220 mya), forming limestone strata. Subsequently, the limestone strata were uplifted by the influence of the Himalayan orogeny after the Late Mesozoic era, which has been eroded by wind and rain for a long time (Clements et al. 2006; Sterling et al. 2006). Thus, Vietnam has vast karst of ~ 60,000 km^2^ (~ 15% of the total area of karst in Southeast Asia) and many limestone caves of different sizes, structures, formation history, and degrees of geographical isolation (Sterling et al. 2006), which are rich in troglobites (Deharveng et al. 2001).

The Cao Bang Province is located in a karst region of northern Vietnam and supports hundreds of caves varying in size and environmental parameters (Sterling et al. 2006). Little is known about the cave millipedes in the Cao Bang Province. Golovatch (2019) recently described several new paradoxosomatid species, namely *Tylopusnguyeni* Golovatch, 2019, *Parasundaninafaillei* Golovatch, 2019, and *Hylomussrisonchaii* Golovatch, 2019 from the same cave as the new species in the current study. These species are completely troglobiotic, with unpigmented bodies, long antennae, and legs. Interestingly, these discoveries resulted from the study of several caves and surveys. Thus, more intensive studies are suggested to reveal more new species.

## Supplementary Material

XML Treatment for Hyleoglomeris halang

XML Treatment for Hyleoglomeris alba
